# Relationship between body mass index and endogenous fibrinolytic status in 600 patients with ST-elevation myocardial infarction

**DOI:** 10.1093/cvr/cvag047

**Published:** 2026-02-27

**Authors:** Joshua H Leader, Gemma Vilahur, Ramzi A Ajjan, Rahim Kanji, Ying X Gue, Mohamed Farag, Diana A Gorog

**Affiliations:** National Heart and Lung Institute, Imperial College, Dovehouse Street, London SW3 6LY, UK; Department of Cardiology, East and North Hertfordshire NHS Trust, Stevenage, UK; Sant Pau Research Institute (IR SANT PAU), Barcelona, Spain; CIBERCV, Instituto de Salud Carlos III, Madrid, Spain; Leeds Institute of Cardiovascular and Metabolic Medicine, University of Leeds, UK; National Heart and Lung Institute, Imperial College, Dovehouse Street, London SW3 6LY, UK; Liverpool Centre for Cardiovascular Science, Liverpool, UK; Freeman Hospital, Newcastle-upon-Tyne, UK; University of Hertfordshire, Hatfield, UK; National Heart and Lung Institute, Imperial College, Dovehouse Street, London SW3 6LY, UK; Department of Cardiology, East and North Hertfordshire NHS Trust, Stevenage, UK; University of Hertfordshire, Hatfield, UK

**Keywords:** Acute coronary syndrome, Endogenous fibrinolysis, Global thrombosis test, Body mass index

**Time of primary review: 25 days**

Impaired endogenous fibrinolysis is a strong, independent risk factor for recurrent cardiovascular events in patients with acute myocardial infarction (AMI),^1,2^ but its determinants are not fully understood.

Obesity, defined as a body mass index (BMI) ≥30 kg/m^2^, is an established risk factor for cardiovascular disease,^3^ partly through its association with traditional risk factors, such as hypertension, diabetes, and dyslipidaemia,^4^ with thrombotic risk mediated through altered metabolic pathways, chronic inflammation, and endothelial dysfunction.^5,6^

Plasminogen activator inhibitor-1 (PAI-1), a key natural inhibitor of fibrinolysis, is mainly secreted from the endothelium and activated platelets but can also be released by other tissues, such as adipose. Obesity has been associated with elevated PAI-1 activity, due to the increased size and frequency of adipocytes as well as enhanced gene expression in adipose cells.^6^ A correlation between BMI and PAI-1 antigen and activity has been reported in individuals without established coronary disease.^7^ However, the relationship between PAI-1 and BMI in individuals with cardiovascular disease is poorly understood.

In a single centre cohort study, we assessed the relationship between BMI and fibrinolytic status in 600 patients with ST-elevation myocardial infarction (STEMI). The cohort comprised of patients enrolled into 3 prospective studies (ClinicalTrials.gov identifier NCT02562690, EudraCT Number: 2018-003299-11 and UK Independent Research Application System ID no. 260786), approved by the UK Health Research Authority and local Research and Development board and conducted according to the principles outlined in the Declaration of Helsinki. The studies used near-identical sampling methods and inclusion/exclusion criteria, ensuring valid cohort assembly without introducing differences in BMI or risk-factor profiles. All patients gave informed consent. Patients on anticoagulation, with coagulopathy, thrombocytopenia, end-stage renal failure, sepsis, alcohol dependence, or in an investigational trial were excluded. Patient demographics, history, medications, routine blood results, weight, and height were recorded on admission, and BMI calculated [weight (kg)/height^2^ (m)]. Individuals were grouped into BMI categories using the World Health Organization classification.^8^

A blood sample was taken from patients with STEMI upon admission, after dual antiplatelet therapy but before angiography or administration of anticoagulation. The first 5 mL blood was used for routine tests including coagulation and hs-CRP, and the next 5 mL non-anticoagulated blood tested using the automated point-of-care Global Thrombosis Test (Thromboquest Ltd., UK),^1^ with measurement started within 15 s of blood draw. Briefly, flowing blood is subjected to high-shear, resulting in platelet activation and activation of coagulation. The time until occlusive thrombus formation (occlusion time) and subsequent restart of flow due to endogenous thrombolysis (lysis time) are recorded. A further 5 mL citrated blood was centrifuged (2300 × g for 10 min) to yield platelet-poor-plasma and PAI-1 antigen level measured with a commercial ELISA kit (ab269373; Abcam, Cambridge, UK) in randomly selected subgroup of 105 patients, chosen to achieve an even distribution of lysis times and roughly equal representation across the five BMI groups (except underweight).

Differences across BMI categories were assessed using the chi-square test for dichotomous variables, with one-way analysis of variance or Kruskal–Wallis test for continuous variables. Quade’s nonparametric ANCOVA was used to compare groups while adjusting for covariates. Spearman’s correlation coefficient was used. Analyses were performed using SPSS v29.0 (IBMCorp), with *P* < 0.05 taken to indicate significance.

In total, 600 patients were recruited (79% male), classified as normal weight (*n* = 181), overweight (*n* = 208), obese with BMI ≥30 kg/m^2^ (*n* = 203), or underweight (*n* = 8). Individuals with higher BMI were younger, more often had diabetes and higher hs-CRP (*Figure 1*).

**Figure 1:**
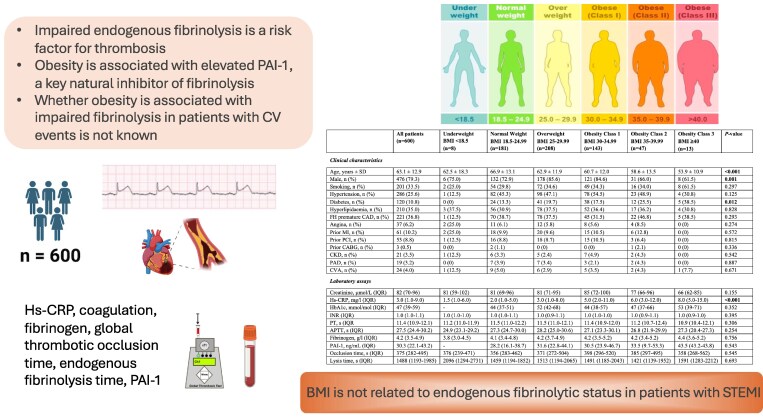
Relationship between endogenous fibrinolysis and body mass index among patients with ST-segment elevation myocardial infarction. Values are presented as n (%) or median (interquartile range, IQR) as indicated. Values in bold are significant (i.e. *P* < 0.05). Abbreviations: AMI, acute myocardial infarction; APTT, activated partial thromboplastin time; BMI, body mass index; CABG, coronary artery by-pass grafting; CKD, chronic kidney disease; CV, cardiovascular; CVA, cerebrovascular accident; FH premature CAD, family history of premature cardiovascular disease; HbA1c, haemoglobin A1c; Hs-CRP, high sensitivity C-reactive protein; INR, international normalized ratio; LT, lysis time; OT, occlusion time; PAD, peripheral arterial disease; PAI-1, plasminogen activator inhibitor 1; PCI, percutaneous coronary intervention; PT, prothrombin time; STEMI, ST-segment elevation myocardial infarction.

BMI was not associated with thrombotic occlusion or endogenous fibrinolysis time, fibrinogen or PAI-1 antigen levels, even after adjustment for covariates, and no associations were observed across sex or diabetes strata or when stratified by BMI. BMI was weakly correlated with hs-CRP (*r* = 0.266, *P* < 0.001) and inversely with prothrombin time (*r* = −0.102, *P* = 0.019). Occlusion time was weakly correlated with hs-CRP (*r* = 0.128, *P* = 0.003) and inversely with platelet count (*r* = −0.106, *P* = 0.01).

Impaired fibrinolysis, found in ∼1 in five patients with STEMI, is a marker of residual cardiovascular risk.^1^ However, our results indicate that BMI is not associated with endogenous fibrinolysis in STEMI patients. Although easy to calculate, BMI is a poor measure of adiposity, since it cannot differentiate between fat mass and lean body mass, and fails to account for variations in body composition related to age, sex, or ethnicity. In healthy individuals, PAI-1 activity correlates modestly with BMI (*r* = 0.4, *P* < 0.001),^9^ and animal studies show that obesity, particularly visceral fat, can increase PAI-1 production, promoting clot formation.^10^ Elevated plasma PAI-1 levels have been associated with excessive visceral rather than subcutaneous fat, particularly central adiposity, suggesting that fat distribution may influence fibrinolytic balance.^6,10^ Waist-to-hip ratio, a more accurate marker of visceral fat distribution, correlated more closely with PAI-1 levels than BMI in obese women.^11^ The contribution of subcutaneous adipose tissue to PAI-1 expression or activity is contentious, although its location may be relevant, with PAI-1 expression in the subcutaneous abdominal depot, but not in femoral fat, influencing circulating PAI-1 levels.^12^

Earlier association of BMI with circulating PAI-1 levels were reported predominantly in healthy individuals and/or in non-acute settings, whereas in our study, measurements were taken during the AMI presentation. As PAI-1 is an acute-phase reactant, measurements during AMI may not reflect the stable/chronic state. Additionally, endothelial cells, hepatocytes and platelets can all contribute substantially to circulating PAI-1 levels.^13^ We did not observe a relationship between PAI-1 level and endogenous fibrinolysis, which could signal that PAI-1 activity may be more important than protein levels during AMI, when PAI-1 may already be maximally activated, or other factors such as fibrin structure and/or plasmin inhibitor levels determine fibrinolytic status. Moreover, a significant proportion of PAI-1 is stored in platelets^13^ and released upon platelet activation, so that circulating PAI-1 level may not reflect the total PAI-1 available to inhibit fibrinolysis, especially at sites of thrombosis.

Limitations include the small number of patients within each BMI band, which were not matched for age or diabetes status. BMI does not capture total adiposity or its distribution, which were not documented and may misclassify cardiometabolic risk. The single measurement of thrombotic status in STEMI may not reflect the stable/chronic state, and antiplatelet medications could have affected fibrinolytic status, although previously were not shown to do so.^1^ Finally, the predominantly Caucasian male population may not be representative of other cohorts.

In conclusion, we found no association between endogenous fibrinolysis and body mass in patients with STEMI. Whether in the chronic state, endogenous fibrinolysis is related to BMI or visceral fat, requires further study.

## Authors’ contributions

J.H.L.—concept and design of the work, analysis of data, interpretation, initial draft of manuscript and revisions. G.V.—interpretation of results, critical review of the manuscript and approving final version. R.A.A.—interpretation of results, critical review of the manuscript and approving final version. R.K.—data acquisition, analysis and critical review. Y.X.G.—data acquisition, analysis and critical review. M.F.—data acquisition, analysis and critical review. D.A.G.—concept and design, critical analysis, interpretation, revisions and final approval.

## Data Availability

The data underlying this article will be shared on reasonable request to the corresponding author.

## References

[1] Farag M, Spinthakis N, Gue YX, Srinivasan M, Sullivan K, Wellsted D, Gorog DA. Impaired endogenous fibrinolysis in ST-segment elevation myocardial infarction patients undergoing primary percutaneous coronary intervention is a predictor of recurrent cardiovascular events: the RISK PPCI study. Eur Heart J 2019;40:295–305. 30380032 10.1093/eurheartj/ehy656

[2] Sumaya W, Wallentin L, James SK, Siegbahn A, Gabrysch K, Bertilsson M, Himmelmann A, Ajjan RA, Storey RF. Fibrin clot properties independently predict adverse clinical outcome following acute coronary syndrome: a PLATO substudy. Eur Heart J 2018;39:1078–1085. 29390064 10.1093/eurheartj/ehy013 PMC6019045

[3] GBD 2015 Obesity Collaborators; Afshin A, Forouzanfar MH, Reitsma MB, Sur P, Estep K, Lee A, Marczak L, Mokdad AH, Moradi-Lakeh M, Naghavi M, Salama JS, Vos T, Abate KH, Abbafati C, Ahmed MB, Al-Aly Z, Alkerwi A, Al-Raddadi R, Amare AT, Amberbir A, Amegah AK, Amini E, Amrock SM, Anjana RM, Arnlov J, Asayesh H, Banerjee A, Barac A, Baye E, Bennett DA, Beyene AS, Biadgilign S, Biryukov S, Bjertness E, Boneya DJ, Campos-Nonato I, Carrero JJ, Cecilio P, Cercy K, Ciobanu LG, Cornaby L, Damtew SA, Dandona L, Dandona R, Dharmaratne SD, Duncan BB, Eshrati B, Esteghamati A, Feigin VL, Fernandes JC, Furst T, Gebrehiwot TT, Gold A, Gona PN, Goto A, Habtewold TD, Hadush KT, Hafezi-Nejad N, Hay SI, Horino M, Islami F, Kamal R, Kasaeian A, Katikireddi SV, Kengne AP, Kesavachandran CN, Khader YS, Khang YH, Khubchandani J, Kim D, Kim YJ, Kinfu Y, Kosen S, Ku T, Defo BK, Kumar GA, Larson HJ, Leinsalu M, Liang X, Lim SS, Liu P, Lopez AD, Lozano R, Majeed A, Malekzadeh R, Malta DC, Mazidi M, McAlinden C, McGarvey ST, Mengistu DT, Mensah GA, Mensink GBM, Mezgebe HB, Mirrakhimov EM, Mueller UO, Noubiap JJ, Obermeyer CM, Ogbo FA, Owolabi MO, Patton GC, Pourmalek F, Qorbani M, Rafay A, Rai RK, Ranabhat CL, Reinig N, Safiri S, Salomon JA, Sanabria JR, Santos IS, Sartorius B, Sawhney M, Schmidhuber J, Schutte AE, Schmidt MI, Sepanlou SG, Shamsizadeh M, Sheikhbahaei S, Shin MJ, Shiri R, Shiue I, Roba HS, Silva DAS, Silverberg JI, Singh JA, Stranges S, Swaminathan S, Tabares-Seisdedos R, Tadese F, Tedla BA, Tegegne BS, Terkawi AS, Thakur JS, Tonelli M, Topor-Madry R, Tyrovolas S, Ukwaja KN, Uthman OA, Vaezghasemi M, Vasankari T, Vlassov VV, Vollset SE, Weiderpass E, Werdecker A, Wesana J, Westerman R, Yano Y, Yonemoto N, Yonga G, Zaidi Z, Zenebe ZM, Zipkin B, Murray CJL. Health effects of overweight and obesity in 195 countries over 25 years. N Engl J Med 2017;377:13–27. 28604169 10.1056/NEJMoa1614362 PMC5477817

[4] Powell-Wiley TM, Poirier P, Burke LE, Despres JP, Gordon-Larsen P, Lavie CJ, Lear SA, Ndumele CE, Neeland IJ, Sanders P, St-Onge MP. Obesity and cardiovascular disease: a scientific statement from the American Heart Association. Circulation 2021;143:e984–e1010. 33882682 10.1161/CIR.0000000000000973 PMC8493650

[5] Morange PE, Alessi MC. Thrombosis in central obesity and metabolic syndrome: mechanisms and epidemiology. Thromb Haemost 2013;110:669–680. 23765199 10.1160/TH13-01-0075

[6] Vilahur G, Ben-Aicha S, Badimon L. New insights into the role of adipose tissue in thrombosis. Cardiovasc Res 2017;113:1046–1054. 28472252 10.1093/cvr/cvx086

[7] Skurk T, Hauner H. Obesity and impaired fibrinolysis: role of adipose production of plasminogen activator inhibitor-1. Int J Obes Relat Metab Disord 2004;28:1357–1364. 15356668 10.1038/sj.ijo.0802778

[8] Obesity: preventing and managing the global epidemic. Report of a WHO consultation. World Health Organ Tech Rep Ser 2000;894:i–xii, 1–253. 11234459

[9] Alessi MC, Nicaud V, Scroyen I, Lange C, Saut N, Fumeron F, Marre M, Lantieri O, Fontaine-Bisson B, Juhan-Vague I, Balkau B, Tregouet DA, Morange PE; DESIR Study Group. Association of vitronectin and plasminogen activator inhibitor-1 levels with the risk of metabolic syndrome and type 2 diabetes mellitus. Results from the D.E.S.I.R. prospective cohort. Thromb Haemost 2011;106:416–422. 21800006 10.1160/TH11-03-0179

[10] Shimomura I, Funahashi T, Takahashi M, Maeda K, Kotani K, Nakamura T, Yamashita S, Miura M, Fukuda Y, Takemura K, Tokunaga K, Matsuzawa Y. Enhanced expression of PAI-1 in visceral fat: possible contributor to vascular disease in obesity. Nat Med 1996;2:800–803. 8673927 10.1038/nm0796-800

[11] Landin K, Stigendal L, Eriksson E, Krotkiewski M, Risberg B, Tengborn L, Smith U. Abdominal obesity is associated with an impaired fibrinolytic activity and elevated plasminogen activator inhibitor-1. Metabolism 1990;39:1044–1048. 2215252 10.1016/0026-0495(90)90164-8

[12] Mavri A, Alessi MC, Bastelica D, Geel-Georgelin O, Fina F, Sentocnik JT, Stegnar M, Juhan-Vague I. Subcutaneous abdominal, but not femoral fat expression of plasminogen activator inhibitor-1 (PAI-1) is related to plasma PAI-1 levels and insulin resistance and decreases after weight loss. Diabetologia 2001;44:2025–2031. 11719834 10.1007/s001250100007

[13] Dellas C, Loskutoff DJ. Historical analysis of PAI-1 from its discovery to its potential role in cell motility and disease. Thromb Haemost 2005;93:631–640. 15841306 10.1160/TH05-01-0033

